# High-Extinction-Ratio Chiral Mid-Wave Infrared Photodetector Using Trapezoidal Si Pillars

**DOI:** 10.3390/mi17020181

**Published:** 2026-01-28

**Authors:** Yingsong Zheng, Longfeng Lv, Yuxiao Zou, Bo Cheng, Hanxiao Shao, Guofeng Song, Kunpeng Zhai

**Affiliations:** 1 Chengdu Entrepreneurship College, Chengdu Polytechnic, 83 Tianyi Street, Chengdu 610041, China; 18109090666@163.com; 2 Institute of Semiconductors, Chinese Academy of Sciences, Beijing 100083, China; lflv@semi.ac.cn (L.L.); sgf@semi.ac.cn (G.S.); 3 Kunming Institute of Physics, Kunming 650223, China; 13307110322@fudan.edu.cn; 4 National Key Laboratory of Infrared Detection Technologies, Kunming Institute of Physics, Kunming 650223, China; 5 Sichuan Provincial Engineering Research Center of Thermoelectric Materials and Devices, Chengdu 610041, China; 6 Institute of Intelligent Photonics, Nankai University, Tianjin 300071, China

**Keywords:** MWIR photodetector, metasurface, circular polarization extinction ratio

## Abstract

Although the polarization state, as a key physical dimension of light, plays an irreplaceable role in many frontier fields such as quantum communication and chiral sensing, traditional photodetectors are limited by the inherent optical isotropy of materials and thus are unable to directly distinguish circular polarization information. This paper numerically reports a miniature circular polarization photodetector based on chiral metasurfaces, which achieves an excellent extinction ratio of up to 31 dB through the collaborative regulation of geometric displacement manipulation and tilt angle operation. This device utilizes the symmetry-breaking effect to construct significantly different transmission spectral responses between left circularly polarized light (LCP) and right circularly polarized light (RCP). Our research not only provides a high-performance implementation solution for on-chip polarization detection but also opens up new paths for the future development of quantum optics, integrated sensing, and ultra-compact polarization optical systems.

## 1. Introduction

Polarization of light, as a fundamental optical degree of freedom of light, carries key information required in fields such as quantum cryptography, biomedical imaging, and remote sensing [1,2,3]. Traditional polarization detection techniques usually rely on bulky discrete optical components such as wave plates and polarizing beam splitters, facing inherent limitations such as complex system alignment and difficulty in integration [4]. In recent years, compact linear polarization photodetectors based on anisotropic nanomaterials (such as two-dimensional perovskites and nanowires) have made significant progress [5,6]. However, due to their intrinsic symmetry, these materials cannot directly distinguish circular polarization states—yet the precise detection of circular polarization is a core requirement in cutting-edge fields such as chiral photonics and spin-optical computing [7].

To address this challenge, metasurfaces [8,9,10,11,12], with their ability to flexibly manipulate light fields at subwavelength scales, offer a promising solution for on-chip polarization optics. Dielectric metasurfaces, through their subwavelength-scale “meta-atom” arrays, can precisely control the phase, amplitude, polarization, and frequency of light in unprecedented ways, providing a powerful platform for achieving compact and efficient photonic devices [13,14,15,16]. In terms of linear polarization detection, a polarization extinction ratio exceeding 15 dB has been achieved by designing metasurfaces with in-plane asymmetry [17]. However, distinguishing between circular polarizations requires metasurfaces with chiral responses [18,19,20,21,22,23], which need to exhibit significant asymmetric transmission characteristics for LCP and RCP. Previous studies, such as designs based on plasmonic helical gratings or multilayer twisted metamaterials, have often been limited by complex multilayer fabrication processes or low efficiency [24,25,26,27,28]. On the other hand, although single-layer metasurfaces based on dielectric materials have achieved excellent circular dichroism, their light typically needs to be incident from the substrate side [29,30,31,32], which is incompatible with the conventional top-integration process of detectors. Therefore, the core challenge lies in designing a single-chip integrated chiral metasurface that combines high extinction ratio, small size, and high efficiency.

Here, we report a compact circularly polarized photodetector (CPPD) that achieves a circular polarization extinction ratio (CPER) of 31 dB at a wavelength of 4.3 μm by monolithically integrating a chiral metasurface with a type-II superlattice detector. By cooperatively regulating the symmetry breaking introduced by geometric displacement and tilt angle, the designed metasurface unit generates highly differentiated transmission responses to left- and right-handed circularly polarized light, with a circular dichroism (CD) exceeding 80%. The chiral metasurface is fabricated using silicon materials that are fully compatible with semiconductor processes and can be directly embedded in standard photodetector fabrication processes. Additionally, this paper systematically analyzes the impact of key process errors on the final performance of the device.

## 2. Materials and Methods

**Figure 1:**
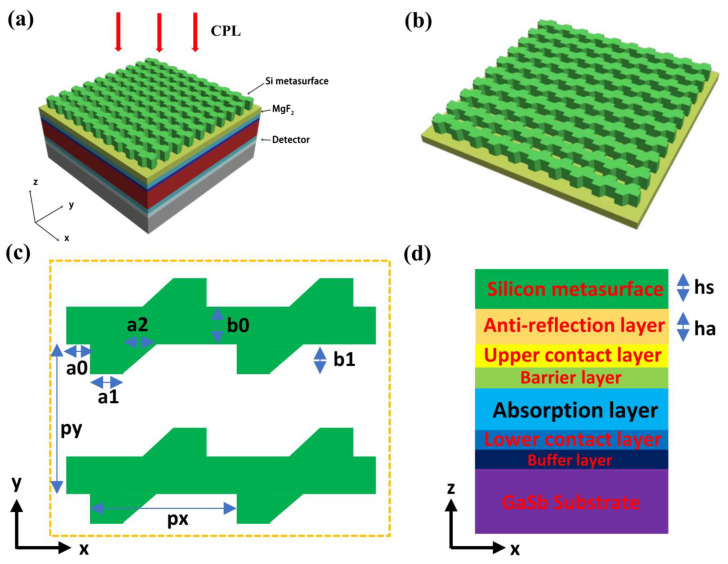
The structure and material information of the mid-wave CPPD. (**a**) Three-dimensional structure diagram of the mid-wave CPPD. The incident light is circularly polarized light (CPL), and the direction of the incident light is the Z-axis. (**b**) Three-dimensional structure diagram of the top chiral silicon metasurface. (**c**) The cross-sectional view of the silicon metasurface in the xy plane. The px = 2.35 μm, py = 2.35 μm, a0 = 0.92 μm, a1 = 2.91 μm, a2 = 1.33 μm, b0 = 2.76 μm, b1 = 1.68 μm. (**d**) The cross-sectional view of the mid-wave CPPD in the xz plane, which can show the thickness and material composition of the device. The hs = 1.61 μm, ha = 2.68 μm.

Figure 1a shows the three-dimensional structure of the mid-wave circularly polarized photodetector, which consists of a type-II superlattice photodetector at the bottom and a chiral silicon metasurface at the top. Figure 1b indicates that the chiral silicon metasurface is composed of a MgF2 film and a silicon pattern array. Figure 1c is the xy cross-sectional view of the silicon metasurface, which shows that the unit cell of the metasurface is composed of two right-angled trapezoids and a rectangular strip. The lower trapezoid can be obtained by rotating the upper trapezoid counterclockwise by 180 degrees. Figure 1d is the xz cross-sectional view of the circularly polarized photodetector, highlighting the functionality of each layer. Additionally, the structure information of the type-II superlattice photodetector from bottom to top is a 500 μm GaSb substrate, a 500 nm AlGaSb buffer layer, a 230 nm P-type doped GaSb, 400 pairs of 2.5 nm InAs/2.5 nm GaSb type-II superlattice absorption layers, a 240 nm AlGaSb barrier layer, and a 500 nm InAs top contact layer. The optical refractive indices of silicon and MgF2 at a wavelength of 4.3 μm are 3.48 and 1.38, respectively. The real part of the refractive index of the absorption layer in the type-II superlattice photodetector at a wavelength of 4.3 μm is 3.8, and the optical absorption length is approximately 1.7 μm. The absorption rate of the device is calculated by commercial software COMSOL 5.6, which is equal to 1 minus the transmission rate minus the reflection rate. Moreover, the specific structure of the optical model is a cube with a typical size of the operating wavelength. Periodic boundary conditions and (perfectly matched layers) are, respectively, applied to the four sides and (top and bottom) surfaces of the cube to reduce the computational load. The polarization information of the incident light is regulated by the incident port. The reflection rate and transmission rate are the area integrals of the Poynting vector at the reflection port and transmission port, respectively.

## 3. Results

**Figure 2:**
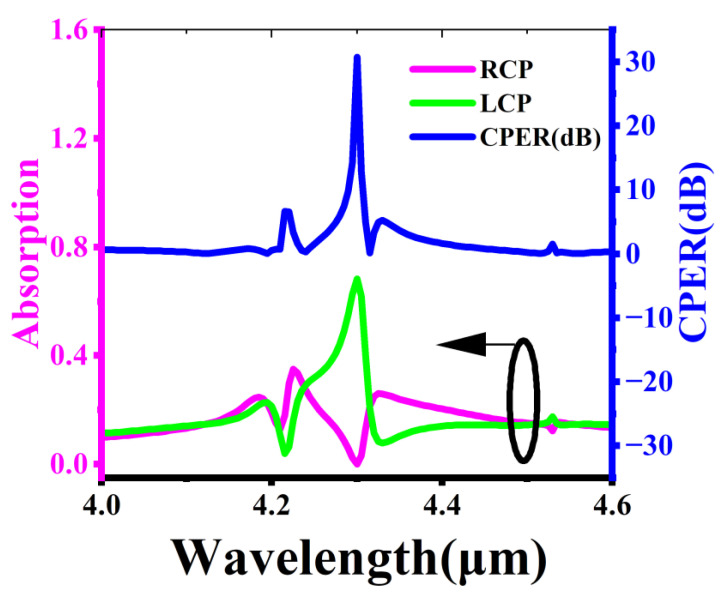
Absorption spectra and CPER spectra of CPPD. The combination of the black circle and arrow in the lower right corner pointing to the left indicates that the two curves below correspond to the left axis of the dual Y-axis.

Figure 2 shows the absorption spectrum and CPER spectrum of CPPD in Figure 1. Here, $CPER=10\times {log}_{10}{(A}_{L}/A_{R})$, where $A_{L}$ and $A_{R}$ represent the optical absorption rates of CPPD under LCP and RCP incident conditions, respectively. The light red curve and the green curve represent the absorption rates, and their corresponding *Y*-axis is on the left. For the RCP incident case, there are three typical absorption valleys in the spectrum within the 4 to 4.6 μm band, located near 4.21 μm, 4.3 μm, and 4.53 μm. The absorption peak at 4.6 μm is extremely weak, almost approaching the failure state where the detector does not respond. For the LCP incident case, when the wavelength is less than 4.23 μm, the spectral line is almost the same as that of RCP, corresponding to an insignificant CD. However, when the wavelength is greater than 4.23 μm, the spectral lines of RCP and LCP are approximately mirror images of each other, corresponding to a significant CD. Additionally, the highest efficiency of CPPD is approximately 0.72. The blue solid line shows the CPER information of CPPD. There are three peaks in the CPER spectrum, but the peak exceeding 10 dB only exists at the operating wavelength of 4.3 μm, corresponding to a super-large 31 dB. The phenomenon that the peak positions of CPER almost correspond one-to-one with the absorption valleys in the RCP absorption spectrum also exists. This is because the value of CPER is usually determined by the small quantity $A_{R}$ in the denominator.

### 3.1. Structural Evolution of the Unit Cell of the Metasurface

**Figure 3:**
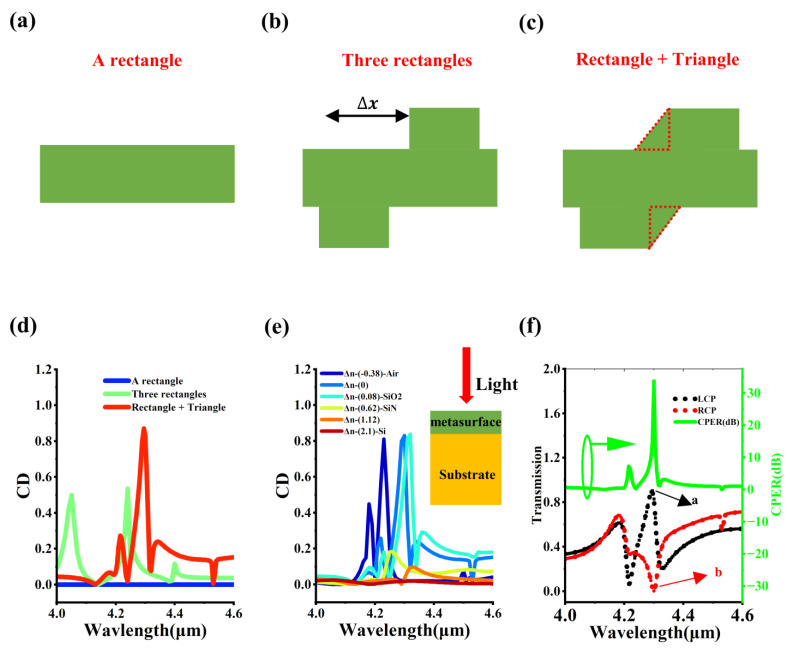
Structural variations and CD spectra. (**a**) a single rectangular unit cell. (**b**) three rectangular unit cells. (**c**) a combination of rectangular and triangular. (**d**) CD spectra of the metasurface corresponding to the three different unit cells. (**e**) CD spectra corresponding to different substrates. (**f**) Transmission and CPER spectra of the optimized structure. The combination of a green arrow and a green circle indicates that CPER corresponds to the right axis of the dual Y-axis. The letters a and b respectively represent the wavelength corresponding to the extreme values of the transmittance.

The geometric unit cell design of chiral metasurfaces is generally a chiral symmetric structure to break mirror symmetry and thereby generate differentiated optical responses when light interacts with the structure, achieving selective control of LCP/RCP. Figure 3a–c shows the entire process of the evolution of the unit cell of metasurfaces from a non-chiral symmetric structure to a chiral symmetric structure. Figure 3a corresponds to the case where the metasurface unit cell is a silicon bar. The silicon bar has C_2_ rotational symmetry and does not belong to a typical chiral symmetric structure. As shown in Figure 3b, when two rectangles are placed on the upper and lower sides of the silicon bar, due to the horizontal misalignment of the two rectangles, the metasurface unit cell becomes a chiral symmetric structure. Figure 3c shows the case where two triangles are added, which can enhance the chiral symmetry. Figure 3d shows the CD spectra of the metasurface under different unit cell structures. It can be found that the intensity of the CD spectrum corresponding to the unit cell in Figure 3a is zero, the peak intensity of the CD peak corresponding to the unit cell composed of the silicon bar and the two rectangles does not exceed 0.6, while the CD intensity corresponding to the combination of the rectangle and the triangle can reach 0.9. This increasing CD intensity is likely due to the introduction of complex geometric structures, which increases the chiral symmetry of the metasurface. Additionally, for dielectric metasurfaces, they generally require a high refractive index difference Δn to enable subwavelength structures to accumulate sufficient phase differences within an extremely short propagation distance, thereby efficiently controlling the light wavefront [33]. Figure 3e shows the influence of Δn on CD. When Δn is greater than 0.62, the metasurface no longer has chiral modulation capability. Here, Δn = $n_{MgF2}$−$n_{S}$, where $n_{MgF2}$ is the refractive index of MgF_2_ and $n_{S}$ is the refractive index of the other substrate. Figure 3f shows the transmission spectrum and CPER spectrum of the metasurface in Figure 3c. The maximum transmission peak of LCP is peak a, with an intensity close to 100%, while the minimum intensity of the RCP valley corresponds to valley b, which is approximately 0. The maximum value of CPER occurs at a wavelength of 4.3 μm, approximately 31 dB. Here, $CPER=10\times log(T_{LCP}/T_{RCP})$ where $T_{LCP}$ and $T_{RCP}$ are the transmission rates of the metasurface for LCP and RCP, respectively.

### 3.2. Analysis of Optical Resonance Modes

**Figure 4:**
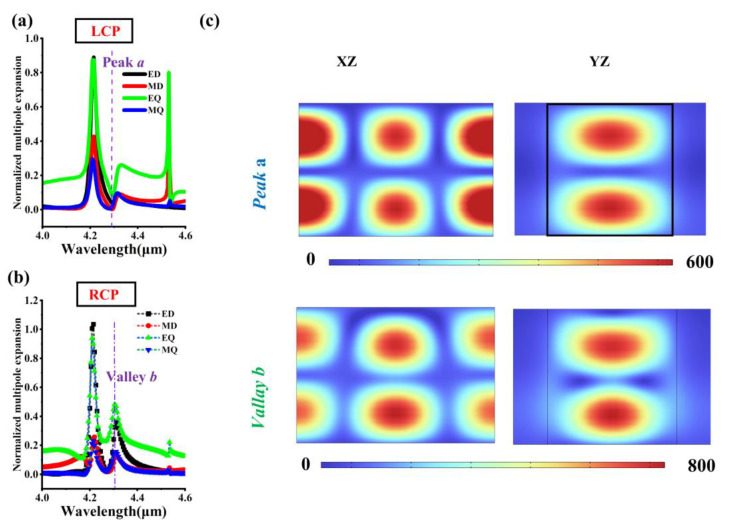
Multipole expansion and magnetic field intensity distribution. (**a**) Multipole expansion of the chiral metasurface under LCP incidence. (**b**) Multipole expansion of the chiral metasurface under RCP incidence. (**c**) Magnetic field distribution of the cross-section of the metasurface at peak *a* and valley *b*. Define peak a as the maximum value of the LCP transmittance near the 4.3 μm wavelength in Figure 3f, and valley *b* as the minimum value of the RCP transmittance near the 4.3 μm wavelength in Figure 3f.

Multipole expansion [34] can precisely reveal the complex electromagnetic resonant modes in metasurface units, which is crucial for understanding and optimizing the interaction between light and subwavelength structures and achieving efficient light field control. The multipole expansion method is a theoretical approach that decomposes the overall electromagnetic response of a complex source (such as a metasurface unit) into the sum of contributions from basic radiation units such as electric dipoles (ED), magnetic dipoles (MD), electric quadrupoles (EQ), and magnetic quadrupoles (MQ). Figure 4a shows the normalized multipole expansion spectrum intensity of the metasurface under LCP incidence. It can be observed that near peak a, the intensities of ED, MD, EQ, and MQ all correspond to a minimum value, which can be simply understood as no resonances occurring within the metasurface. This extremely weak resonance phenomenon may imply that at this time, the metasurface can be equivalently regarded as a dielectric film with an impedance matching air, corresponding to a transmission peak. Figure 4b shows the multipole expansion spectrum of the metasurface under RCP incidence, where the intensities of ED and EQ are most significant at valley b. It can be considered that the destructive interference of the ED and EQ modes leads to a transmission valley close to 0. Figure 4c presents the magnetic field intensity distribution of the metasurface at peak a and valley b. Regardless of whether the cross-section is the XZ plane or the YZ plane, there are significant red spheres appearing within the metasurface, indicating that a standing wave effect of the guided mode resonance type has occurred, which explains the extreme values of the spectral transmittance.

### 3.3. Analysis of Vertical Coupling Effect

**Figure 5:**
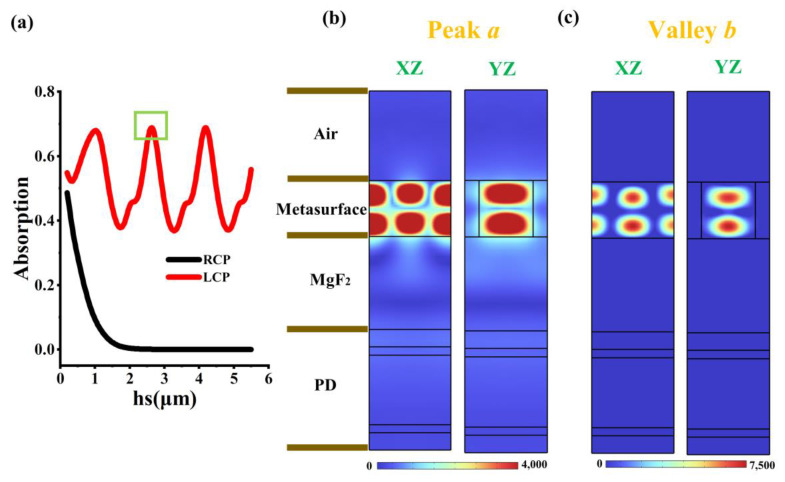
(**a**) The influence of the thickness hs of the dielectric layer between the chiral metasurface and the detector on the CPPD. The green box refers to the best hs. (**b**) The magnetic field distribution diagram of the CPPD at the peak a. (**c**) The magnetic field distribution diagram of the CPPD at valley *b*.

To ensure the efficient transmission of 4.3 μm wavelength light to the detector, it is necessary to simulate the thickness of the MgF_2_ anti-reflection layer between the chiral metasurface and the detector to guarantee the minimum coupling between them. Figure 5a shows the influence of the MgF_2_ film thickness *hs* on the absorption rate of the CPPD. For the case of RCP incidence, the absorption rate first decreases and then remains constant as *hs* increases. For the case of LCP incidence, the absorption rate shows periodic oscillations as *hs* increases. Additionally, considering the requirements for the quality of the MgF2 film formation, *hs* cannot be too large. Ultimately, an optimal *hs* of 2.68 μm is marked by a green box. Figure 5b shows the magnetic field intensity of the CPPD at peak *a*. It can be found that the magnetic field can penetrate the metasurface and enter the detector below. However, as shown in Figure 5c, when the incident light corresponds to valley *b*, the metasurface exhibits the characteristics of a perfect reflector, prohibiting any photons from entering the detector.

### 3.4. Analysis of Potential Errors

**Figure 6:**
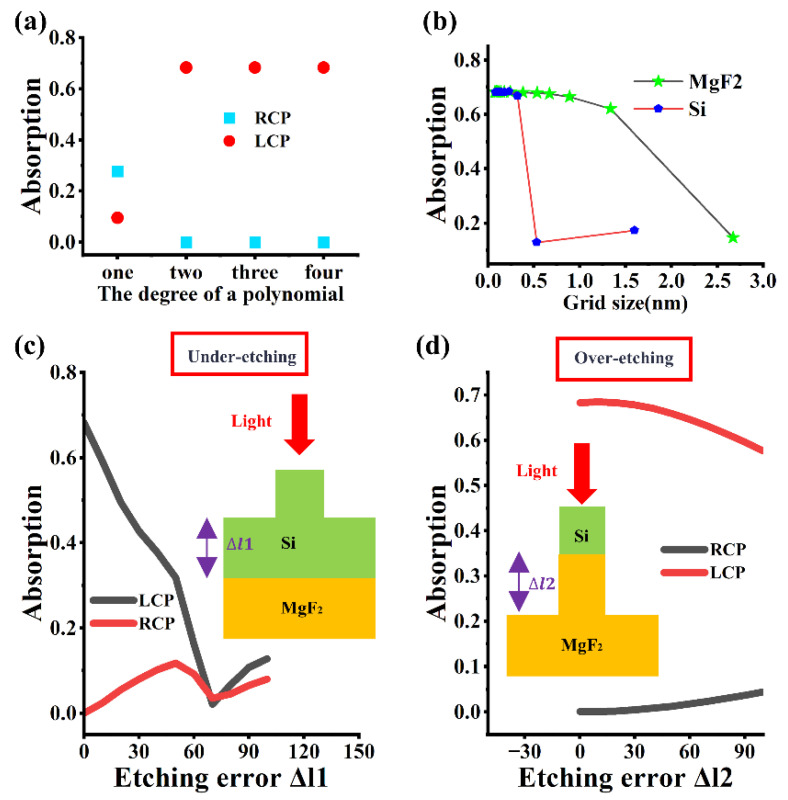
The influence of (**a**): polynomial degree, (**b**): mesh size, (**c**): under-etching depth error, (**d**): over-etching depth error.

In addition to considering the influence of MgF_2_ film thickness, CPPD also needs to take into account other factors, such as the degree of the polynomial in the optical model, mesh size, under-etching depth error, and over-etching depth error, etc. Figure 6a shows the influence of the degree of the polynomial function in the electromagnetic finite element algorithm on the absorption rate. When the degree of the polynomial is greater than or equal to 2, the calculation results of COMSOL tend to be stable. Figure 6b shows the influence of the mesh size of MgF_2_ and silicon on the CPPD. It is obvious that when the mesh size is less than 40 nm, the simulation results are convergent. However, for silicon metasurfaces, large mesh sizes will cause significant result fluctuations. Figure 6c,d, respectively, show the influence of under-etching depth error and over-etching depth error on the device. Both cases have significant damage effects. Especially for the under-etching situation, when the etching error exceeds 60 nm, the device almost loses its chirality. Therefore, in the formal processing of the device, the etching rate and time can be precisely controlled, high-selectivity etching processes can be adopted, and real-time endpoint detection can be used to ensure that the etched structure size is as consistent as possible with the design target, thereby achieving the ideal etching state.

## 4. Conclusions

In summary, we report a CPPD with a simple fabrication process. This device achieves a circular polarization extinction ratio of up to 31 dB at a wavelength of 4.3 μm by monolithically integrating a precisely designed chiral silicon metasurface with a type-II superlattice absorber. Our design constructs a metasurface with a strong chiral response by regulating the displacement and tilt angle of the structural units, resulting in highly differentiated transmission spectra for LCP and RCP, with a circular dichroism exceeding 80%. Moreover, the all-dielectric metasurface is fabricated using a technology compatible with complementary metal-oxide-semiconductor processes, providing an expandable path for the development of chip-level polarization detection systems. The design principles revealed in this work can be further extended to other spectral bands by adjusting the metasurface configuration and absorption materials, thus opening up new possibilities for wide-spectrum detection of all Stokes parameters.

## Data Availability

The raw data supporting the conclusions of this article will be made available by the authors on request.
